# The Book World of Medicine and Science

**Published:** 1893-04-08

**Authors:** 


### The Book World of Medicine and Science.

### Book Reviews.

National Life and Character. A Forecast. By Charles H. Pearson. (Macmillan and Co.)

In this volume the author "professes to consider what is likely to happen if we go on for two hundred years more as we have gone on for the last three-quarters of a century." This forecast is made in a spirit very unusual among those who guess at the future, of temperate and philosophic consideration. While pointing out the probability that the lower races may eventually predominate in the population of the world, and this by the direct action of the benefits of our civilisation introduced among them, Mr. Pearson thinks the consolation must be that the changes have been inevitable, and that when we are thrust aside by peoples whom we looked down upon as servile, we are to remember that it has been our work to carry peace and law and order over the world, that others may enter in and enjoy. It is not exactly an exhilarating outlook, and when we are confronted with the picture of the dense population of England, confined to its small territory by the discouragement in every country of immigration, the author can only hazard the prediction "in that case there must be some means of supporting the ever-pressing burden of fresh lives." The very interesting chapter on the dangers of political development, discusses, among other points, the *pros* and *cons* of city life. Admitting that there is no conclusive evidence to show that the race of townsmen degenerate, Mr. Pearson urges that "we have to take into account that the great English and Scotch towns have been draining the life blood of the country districts for more than a century. So long as this lasts and is on a large scale we are not in a position to appreciate how far town life tells upon the physique of the people. Not only do the vigorous countrymen replenish the towns with new life, but they have a tendency to crowd out and starve the weaker and more stunted specimens of humanity, who are the outcome of several generations that have grown up without access to light and air, and without muscular exercise. It must be admitted that the average of life in England is now longer than it was—a fact which seems inconsistent with reduced vitality. Still, it may be questioned if we can infer more from this than that science to some extent compensates the disadvantages that result from the city life carried to excess." In his visions of the future the author lays great stress on the probable prolongation of life. "If we can conceive cholera, typhus, and all such diseases extirpated by sanitary science, the diseases that scourge immorality disappearing before a rigid police, and cures such as are even now anticipated, actually discovered for consumption and cancer, perhaps even for diseases of the liver and kidneys, it will not be too much to say that the present expectation of life under healthy conditions would be more than doubled." Yet even here the outlook is not unmitigatedly hopeful, for the prospect of a world in which the term of life is sensibly prolonged, and which, therefore, becomes "predominantly a world of old people," is not without drawbacks. It will be seen that the book is more a statement of problems than an attempt to solve them. It is a book which will be widely read and discussed, and which in its very inconclusiveness displays the author's firm grasp of the subjects he handles.

THE BOOK WORLD OF MEDICINE AND SCIENCE.—(*continued*).

### FIRST IMPRESSIONS OF BOOKS RECEIVED.

[It is requested that all books intended to be noticed in this column may be sent to the Editor, at the Office, 428, Strand, W.C., not later than Tuesday evening in each week.]

BIOGRAPHICAL HISTORY OF GUY'S HOSPITAL. By SAMUEL WILLS, M.D., LL.D., F.R.S., and G. T. BETTANY, M.A., B.Sc. (Ward, Lock, Bowden, and Co., London, 1892.

The history of any one of our great hospitals is full of interest, of none is this more true than of Guy's. The late Mr. Bettany has discovered much that was before unknown about the pious founder of this great charity, and has saved his name and memory from unjust slurs that have been cast upon it. But the part of the volume which appeals to us most is that devoted to memories of the great physicians and surgeons of Guy's. In these pages Astley Cooper lives again, and the vigorous and faithful sketches of later men such as Gull and Moxon, will be very welcome to those who were privileged to know them.

OUR TEMPERAMENTS: THEIR STUDY AND THEIR TEACHING. A Popular Outline. By A. STEWART, F.R.C.S., Edin. Second Edition. (Crosby Lockwood and Son.)

The author's aim is to familiarise us with the true use of the word "temperament," and of the physical characteristics of the great types of temperament. He therefore deals with a subject often touched upon by medical writers, but rarely dealt with with precision. Latterly, when medicine has become to some extent an exact science, the tendency has been to pay little heed to such a matter as temperament, which is so far removed in most people's mind from either exactness or science. Mr. Stewart deals with the question from its general aspect rather than its medical, and he shows how a knowledge of temperament is of great value in education and in the planning of a life. A very interesting book.

DISEASES OF THE SKIN: Their Description, Pathology, Diagnosis and Treatment, with Special Reference to the Skin Eruptions of Children, and an Analysis of 12,000 Cases of Skin Disease. By H. RADCLIFFE CROCKER, M.D., Lond., F.R.C.P.; Physician for Diseases of the Skin in University College, London. Second Edition. (London: H. K. Lewis.)

We are glad to see that a second edition of Dr. Crocker's admirable work has been called for. It is the best work on the subject in the English language, far removed from the "manual" or "companion" type of which many treatises on diseases of the skin are examples. The work is done with the utmost care and fulness, and for all who want a thorough, reliable, and complete treatise on dermatology, as well as an admirable guide in treatment, we can strongly recommend Crocker's work.

ON SERPENT-WORSHIP, AND ON THE VENOMOUS SNAKES OF INDIA. By SIR JOSEPH FAYRER, K.C.S.I., LL.D., M.D., F.R.S.

This is a paper read before the Victoria Institute by the highest authority on the subject. On serpent-worship there is much more to be said than Sir Joseph Fayrer goes into. The question of venomous snakes is a very important one in India, where 20,000 lives are annually lost from this cause. Sir Joseph Fayrer urges the enormous importance of teaching the natives to recognise the poisonous varieties, and to give rewards for killing only these. The pamphlet contains a report of the discussion which followed the reading of the paper, as well as a special statement by Dr. Augustus Morellen.

